# The old second messenger cAMP teams up with novel cell death mechanisms: potential translational therapeutical benefit for Alzheimer’s disease and Parkinson’s disease

**DOI:** 10.3389/fphys.2023.1207280

**Published:** 2023-06-19

**Authors:** Tong Zhang, Minh D. A. Luu, Amalia M. Dolga, Ulrich L. M. Eisel, Martina Schmidt

**Affiliations:** ^1^ Department of Molecular Pharmacology, University of Groningen, Groningen, Netherlands; ^2^ Department of Molecular Neurobiology, Groningen Institute for Evolutionary Life Sciences, University of Groningen, Groningen, Netherlands; ^3^ Groningen Research Institute for Asthma and COPD, GRIAC, University Medical Center Groningen, University of Groningen, Groningen, Netherlands

**Keywords:** cAMP, oxidative stress, mitochondria, parthanatos, ferroptosis, Alzheimer’s disease, Parkinson disease

## Abstract

Alzheimer’s disease (AD) and Parkinson’s disease (PD) represent the most prevalent neurodegenerative disorders severely impacting life expectancy and quality of life of millions of people worldwide. AD and PD exhibit both a very distinct pathophysiological disease pattern. Intriguingly, recent researches, however, implicate that overlapping mechanisms may underlie AD and PD. In AD and PD, novel cell death mechanisms, encompassing parthanatos, netosis, lysosome-dependent cell death, senescence and ferroptosis, apparently rely on the production of reactive oxygen species, and seem to be modulated by the well-known, “old” second messenger cAMP. Signaling of cAMP via PKA and Epac promotes parthanatos and induces lysosomal cell death, while signaling of cAMP via PKA inhibits netosis and cellular senescence. Additionally, PKA protects against ferroptosis, whereas Epac1 promotes ferroptosis. Here we review the most recent insights into the overlapping mechanisms between AD and PD, with a special focus on cAMP signaling and the pharmacology of cAMP signaling pathways.

## Introduction

Alzheimer’s disease (AD) and Parkinson’s disease (PD) represent two of the most prevalent neurodegenerative disorders worldwide, affecting over 50 million and 10 million people, respectively (Rocca, 2018; Nichols et al., 2022). AD primarily affects learning and memory processes, and is characterized by progressive memory loss, language difficulties, disorientation, mood and behavior changes, and difficulty with daily routine tasks (Long and Holtzman, 2019; Scheltens et al., 2021). PD mainly affects coordination of movements, causing rest tremor, rigidity, bradykinesia, and difficulty with balance and coordination (Tolosa et al., 2021). Although PD can also cause non-motor symptoms such as depression, anxiety, sleep problems, and cognitive impairment, these symptoms are generally less severe than those of AD (Tolosa et al., 2021). AD and PD are multifactorial disorders that seemingly involve multiple cellular pathways and mechanisms. In AD, aggregation of amyloid beta (Aβ) and hyperphosphorylated tau are important contributors to the disease, along with oxidative stress, neuroinflammation, loss of cholinergic neurons, and mitochondrial dysfunction. On the other hand, the development and progression of PD also involve several cellular changes, including accumulation of α-synuclein, dopaminergic neurodegeneration, oxidative stress, neuroinflammation and mitochondrial dysfunction (Hou et al., 2019). Interestingly, accumulated α-synuclein was found in more than 50% of autopsied AD brain while tau protein could also be detected in one-third of PD cases, which may suggest overlapping mechanisms between AD and PD (Roberts et al., 2017; Zhang et al., 2018; Smith et al., 2019; Twohig and Nielsen, 2019; Visanji et al., 2019).

**FIGURE 1:**
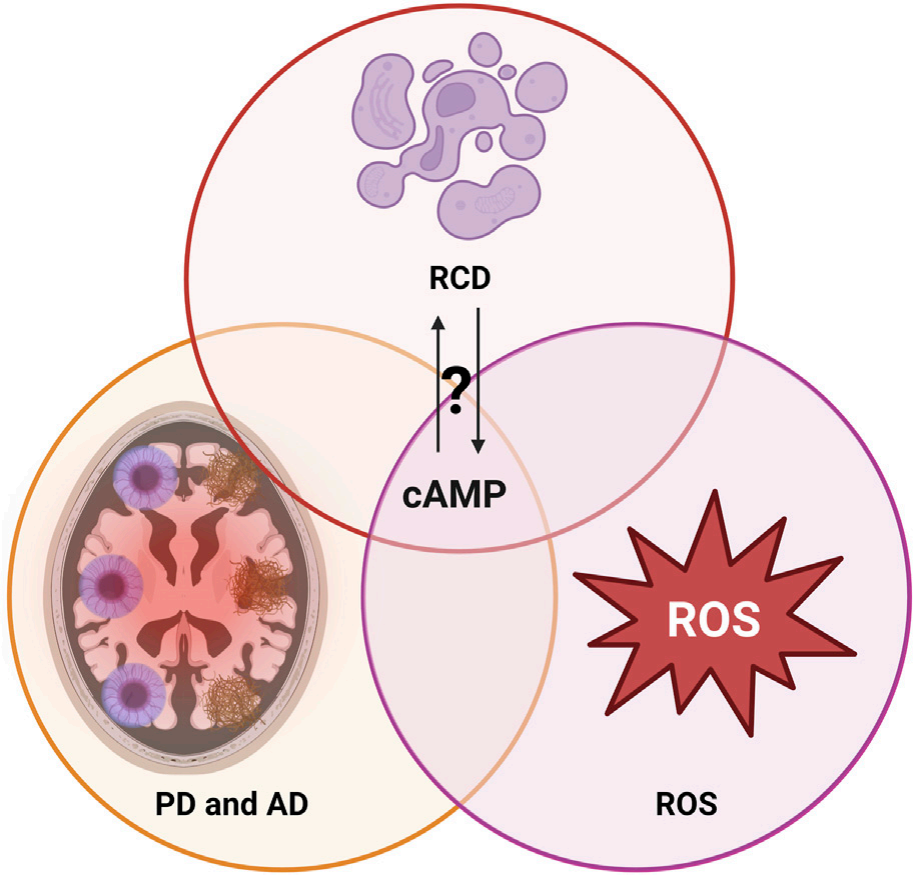
Interplay of regulated cell death (RCD) mechanisms, reactive oxygen species (ROS) and the neurodegenerative disorders Alzheimer’s disease (AD) and Parkinson disease (PD): Mechanisms potentially overlapping on the level of cAMP. For further details, see text.

Reactive oxygen species (ROS), such as superoxide anion (O_2_-), hydrogen peroxide (H_2_O_2_) and hydroxyl radical (-OH), are intermediates in the process of aerobic metabolism. Mitochondria are the main organelles to generate ROS within the cells, particularly during aerobic respiration involving electron transfer through respiratory chain complexes I and III. Low levels of ROS function as signaling molecules, regulating transcription, phosphorylation and other pathways (Mittler, 2017). Some studies suggest that a low level of ROS plays specific role in cellular proliferation, differentiation and immunity (Cairns et al., 2011; West et al., 2011; Mittal et al., 2014; Diebold and Chandel, 2016; Mittler, 2017). In AD and PD, however, most probably due to an insufficient elimination and/or overproduction of ROS aberrantly high cellular ROS levels initiate cellular alterations defined as oxidative stress. For example, under normal physiological conditions, the intracellular level of H_2_O_2_ is tightly controlled within the low nanomolar concentration of approximately 1–10 nM and serves as redox signaling, while concentrations above 100 nM are considered as oxidative stress (Sies and Jones, 2020) (Figure 1). Oxidative stress damages (large) molecules including lipids, proteins and DNA, and thereby impacts cellular activities such as synaptic function, calcium homeostasis, receptor trafficking, endocytosis and regulated cell death (RCD) (Halliwell, 2006; Tönnies and Trushina, 2017). Indeed, increasing evidence indicates that the presence of oxidative stress in brains is closely associated with pathological hallmarks of AD and PD (Sultana et al., 2009; Padurariu et al., 2010; Rajkumar et al., 2019; Trist et al., 2019; Oun et al., 2022). In AD, Aβ oligomers trigger a significant influx of Ca^2+^ into the mitochondria, subsequently stimulating oxidative phosphorylation, and ultimately resulting in dysfunctional mitochondria and increased ROS production. Interestingly, a recent study reviewing RNA sequencing data also indicates that dysfunctional mitochondria in late AD exhibit an alteration of the expression of ROS-related gens in neurons, such as oxidative phosphorylation and electron transport chain (Marmolejo-Garza et al., 2022). In addition, in PD models it has been hypothesized that binding of α-synuclein to mitochondria disrupts the mitochondrial membrane potential, subsequently leading to ROS generation and impairment of mitochondria protein import (Krabbendam et al., 2018). Apart from dysfunctional mitochondria, accumulation of bioactive metals and activated microglia are the other two sources of oxidative stress in AD and PD (Tönnies and Trushina, 2017). Bioactive metals including copper, iron, and zinc, are found to be accumulated in the brains of AD and PD and serve as catalysts in ROS production via Fenton and Haber-Weiss reactions. In addition, in AD and PD, disease-associated microglia (DAM) are activated via damage-associated molecular patterns (DAMPs) that are released in response to neuronal damage or protein aggregation. Activated DAM results in persistent inflammation and ROS production, and have been implicated in the exacerbation of neurodegeneration (Calsolaro and Edison, 2016; Simpson and Oliver, 2020).

**FIGURE 2:**
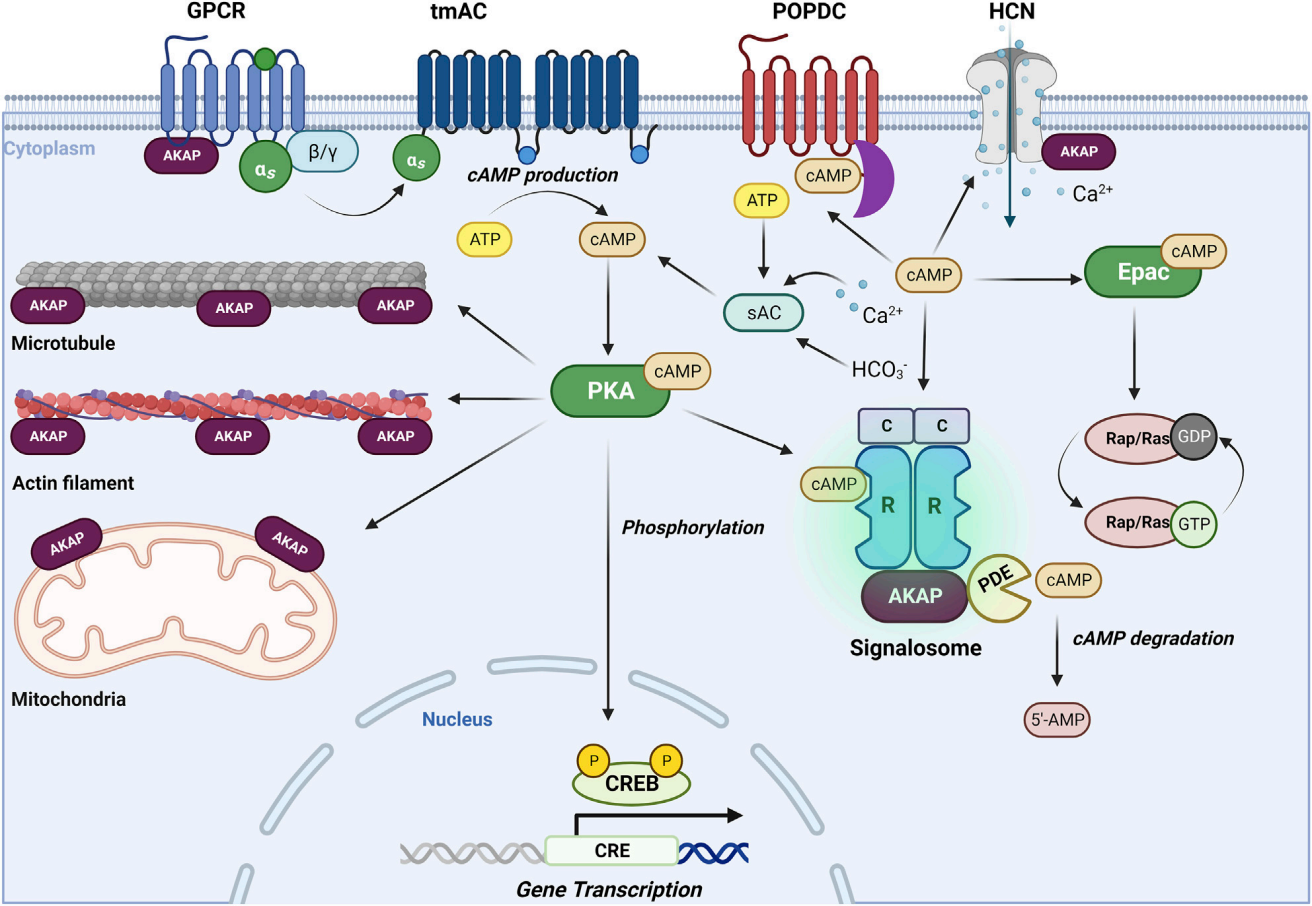
The cAMP signaling pathways. The well-known, “old” second messenger cAMP is generated upon activation of G_s_-coupled receptors (such as the β-adrenoceptors) and subsequent stimulation of a diverse subset of adenylyl cyclases (ACs), shown are the transmembrane ACs (tmACs) and the soluble ACs (sACs). cAMP exerts its effects through its primary effector protein kinase A (PKA), next to the exchange protein directly activated by cAMP (Epac) cyclic nucleotide-gated channels (HCN) and Popeye domain containing (POPDC) proteins (Brand, 2018; Robichaux and Xiaodong Cheng, 2018). The multifaceted signaling properties of cAMP are controlled by A-kinase anchoring proteins (AKAPs) and phosphodiesterases (PDEs), enabling cAMP signaling in space and time (Schmidt et al., 2013; Poppinga et al., 2014; Han et al., 2015). For further details, see text.

Cyclic adenosine monophosphate (cAMP) is a key second messenger that mediates a variety of physiological processes, including memory and learning, myocardial relaxation and contraction, inflammation and immune function (Cheng et al., 2008; Serezani et al., 2008), and various cellular activities, such as calcium homeostasis, gene transcription, glutaminergic receptor trafficking, cell death, and neurotransmission (Schmidt et al., 2013; Liu et al., 2016). However, dysregulation of cAMP signaling has been linked to various diseases such as cancer, diabetes, and neurological disorders, highlighting the importance of tight regulation of intracellular cAMP levels. cAMP signaling is initiated by activation of G protein-coupled receptors (GPCRs), such as the Gs-coupled β-adrenoceptors. Subsequently, Gs activates transmembrane adenylate cyclases (tmACs), leading to an elevation of intracellular cAMP levels. In addition, soluble AC (sAC), known to be regulated by CO_2_/HCO^−3^/pH-, calcium and ATP, contributes to cAMP elevations (Wiggins et al., 2018). The duration and amplitude of cAMP signaling is tightly controlled by phosphodiesterases (PDEs), including cAMP specific PDE4, PDE7, and PDE8, and cAMP/cGMP dual substrate specific PDE1, PDE2, PDE3, PDE10 and PDE11 (Zuo et al., 2019). cAMP signaling pathway involves four main effectors, namely, protein kinase A (PKA), exchange protein directly activated by cAMP (Epac), cyclic nucleotide regulated ion channels and Popeye domain containing (POPDC) proteins, which all share a conserved cAMP binding domain except POPDC proteins (Brand, 2018; Robichaux and Xiaodong Cheng, 2018). As the most studied cAMP effector, PKA exerts its effects by either directly activating proteins or modulating other kinase pathways through phosphorylation. The multifunctional facets of PKA signaling are coordinated by the interaction with A-kinase anchoring proteins (AKAPs), tethering PKA to receptors (such as the Gs-coupled receptors, ion channels) and subcellular structures such as actin filaments, microtubules, mitochondria and nuclei (Poppinga et al., 2014; Han et al., 2015) (Figure 2). PKA also plays a role in regulating gene expression by phosphorylating cAMP response element-binding protein (CREB) in the nucleus. Epac, on the other hand, functions as a guanine nucleotide exchange factor (GEF) and modulates downstream targets by activating small GTPases such as Rap and Ras (Schmidt et al., 2013). Meanwhile, cAMP-gated ion channels, such as hyperpolarization-activated cyclic nucleotide-gated channels (HCNs), directly modulate ion influx and membrane depolarization, facilitating action potentials (Santoro and Shah, 2019). Unlike PKA and Epac, POPDC proteins are transmembrane proteins that interact with cAMP and downstream proteins via their highly conserved intracellular POPEYE domains (Tucker and Zorn, 2022). As mentioned above, AKAPs play a crucial role in cAMP signaling by coordinating G proteins, ACs, PDEs, PKA, and Epac (Nijholt et al., 2008; Schmidt et al., 2013) (Figure 2). This coordination leads to the formation of signalsomes, enabling efficient sensing and functioning of cAMP in various subcellular locations.

Many studies have investigated the effects of ROS on cAMP signaling pathway. For example, in rat adipocytes it was reported that micromolar concentrations of H_2_O_2_ inhibited type II PKA activity via the formation of disulfide bonds between Cys-97 in the regulatory subunit and Cys-199 in the catalytic subunit (De Piña et al., 2008). Similarly, a study in pancreatic islets also suggested that ROS reduced cAMP levels via ROS sensitive ACs and inhibit PKA via oxidizing specific residues (Li et al., 2012). In addition, it was found that ROS can bypass cAMP stimulation and directly modulate PKA activity. In cardiomyocytes, it was reported that ROS oxidized the regulatory subunits of type I PKA at Cys17 and Cys38, promoting disulfide bond formation between two regulatory subunits leaving PKA activity unchanged. The cytosolic disulfide bonds containing type I PKA was subsequently translocated to the membrane and activated independent of cAMP elevations (Brennan et al., 2006). In a model of ischemic heart failure, it was found that increased ROS activated PKA in the absence of cAMP elevations and consequently initiated substrate phosphorylation (Andre et al., 2013). A more recent study in mouse cardiomyocytes addressed that oxidized type I PKA preferentially localized to the lysosome via increased AKAP binding (Simon et al., 2021). On the other hand, the cAMP/PKA pathway can seemingly modulate ROS production by regulating enzyme activities involved in the respiration. It was reported that activation of PKA attenuated ROS production via phosphorylation on NADH ubiquinone oxidoreductase subunit S4 (NDUFS4) in complex I in different human and murine cells (Piccoli et al., 2006). Another study revealed that activation of PKA by forskolin increased complex I activity, leading to a reduction in ROS levels in cancer cells (Palorini et al., 2013). On the contrary, however, in cardiomyocytes, PKA activation triggered by a β-adrenergic agonist promoted ROS production, while pretreatment with a PKA inhibitor abrogated ROS production, suggesting that PKA exerts distinct, cell type specific functions in cellular ROS production (Andersson et al., 2011). In line with these findings, the other main cAMP effector, Epac has been proposed to negatively regulate ROS production. It was reported that Epac downregulated downstream PI3K/Akt pathway, leading to a reduced ROS levels in pancreatic β-cells (Mukai et al., 2011). In another study of ischemia-reperfusion injury, activation of Epac-Rap1 pathway reduced the production of mitochondrial superoxide and thereby prevented ROS production (Stokman et al., 2014). These studies demonstrate the intricate interplay between ROS and cAMP signaling, which depends on several factors, including ROS concentrations, specific cell types and signaling pathways (Figures 1, 2).

In AD, cAMP levels are generally reduced, although sometimes elevated or unchanged, depending on the expression/activity of AC and PDE in specific brain regions, which may contribute to AD pathogenesis (Kelly, 2018; Di Benedetto et al., 2021). The basal, Gαs-mediated, and forskolin-induced AC activities are decreased in the hippocampus and temporal cortex, due to reduced expression of AC1 and AC2, rather than other AC isoforms. However, Gαs-mediated AC activity is only decreased in the cerebellum and occipital cortex (Kelly, 2018). On the other hand, in late AD the mRNA level of PDE4A is reduced in the frontal cortex and CA2, while PDE4D is increased in the putamen (Pérez-Torres et al., 2003; Ugarte et al., 2015). The mRNA level of PDE7A is decreased in the dentate gyrus (DG), whereas the mRNA level of PDE8B is increased in both the hippocampal DG and CA2 regions (Pérez-Torres et al., 2003). Notably, studies in *in vivo* AD models have demonstrated that hippocampal impairments can be mitigated by increasing cAMP levels through either forskolin-stimulated AC activity or reduced expression/activity of PDEs (Kelly, 2018). Furthermore, a number of PDE4 inhibitors have shown clinical potential to improve cognition and disease severity via compensating decreased cAMP in AD (Prickaerts et al., 2017). In addition, soluble Aβ was found to lead to mitochondrial Ca^2+^ overload (Calvo-Rodriguez et al., 2020), which may trigger activation of mitochondrial sAC in AD (Valsecchi et al., 2014). Studies in *in vivo*, *in vitro* models of AD and AD patients reported on reduced expression and/or activity of global PKA (Bonkale et al., 1996; Kelly, 2018). It is worth noting that localized PKA and AKAP5 are involved in hyperphosphorylation of tau pathology in AD (Jicha et al., 1999). Localized PKA signaling may provide an explanation for the puzzling finding that rather low concentrations of the PKA inhibitor H89 attenuated memory deficits, oxidative stress and NfkB signaling in the male Wistar rats (Eftekharzadeh et al., 2012; Amini et al., 2015). In PD, on the other hand, alterations in cAMP due to (gene and protein) changes in PDE4D, PDE8B and PDE10A correlates with dementia progression and symptom severity (Azuma et al., 2015; Niccolini et al., 2015; Kaut et al., 2017). Similarly, in the 6-hydroxydopamine (6-OHDA) model of PD a decreased PDE10A expression correlated with an increased cAMP level (Sancesario et al., 2014). Signorile et al. (2021) performed studies in fibroblasts from Parkin-mutants and reported on cAMP elevations due to reduced PDE4 activity, while treatment with resveratrol (known to inhibit several molecular targets next to PDE4) restored the cellular cAMP level pointing to the involvement of cAMP modulators distinct from PDE4. Indeed, Signorile et al. (2021) demonstrated that resveratrol inhibited transmembrane ACs (tmACs) and activated soluble ACs (sACs) via upregulation of cytosolic and mitochondrial Ca^2+^, resulting in a remodeling of the cAMP homeostasis. In addition, compartmentalized cAMP/PKA also play an important role in PD pathology. A study revealed that both AKAP1 overexpression and PKA activation restored dysfunctional mitochondrial respiration and lysosome function in PD cell models with deficient PTEN-induced kinase 1 (PINK1), indicating the role of compartmentalized cAMP/PKA signaling in mitochondrial pathology (Dagda et al., 2011).

Extensive evidence has already indicated that cell death can occur in either accidental ways or regulated ways. Compared with accidental cell death, regulated cell death (RCD) is tightly and precisely modulated by various molecular machineries, and it has been suggested that RCD can be inhibited genetically and/or pharmacologically (Galluzzi et al., 2018). The first identified RCD, apoptosis, was found by John Kerr in 1972 and had been used as the synonyms for all RCDs up until 2000 (Kerr et al., 1972). After 2000, many novel RCD mechanisms, such as parthanatos, NETosis, lysosome-dependent cell death, cellular senescence and ferroptosis, were gradually identified and studied (Tang et al., 2019).

Mature neurons in a healthy CNS are generally resistant to cell death and can persist throughout the individual lifespan (Chi et al., 2018). Neuronal cell death is generally observed as a final outcome of multiple stress accumulations, surpassing the ability of neurons to recover. In AD, cell death occurs in various regions, including the entorhinal cortex, nucleus basalis, and locus coeruleus, even before clinical symptoms (Arendt et al., 2013). As AD progresses, more severe neuronal loss occurs in the frontal cortex and other subcortical regions (Martínez-Pinilla et al., 2016). In PD, over 50% of neurons in the substantia nigra are lost prior to diagnosis (Ross et al., 2004). Until now, various RCDs are observed in AD and PD, and we provide here the most updated summary. However, it remains unclear whether RCDs are primary contributors or merely represent secondary responses due to the underlying pathophysiology of AD and PD. Oxidative stress is a well-established trigger for various RCDs, as comprehensively reviewed in (Tang et al., 2019). cAMP signaling, on the other hand, has been demonstrated to engage in reciprocal regulation with oxidative stress. In this review, we aim to elaborate several ROS related RCD mechanisms in AD and PD, and will emphasize the impact of cAMP signaling to those novel RCD mechanisms (Figures 1, 2).

### Parthanatos

**FIGURE 3:**
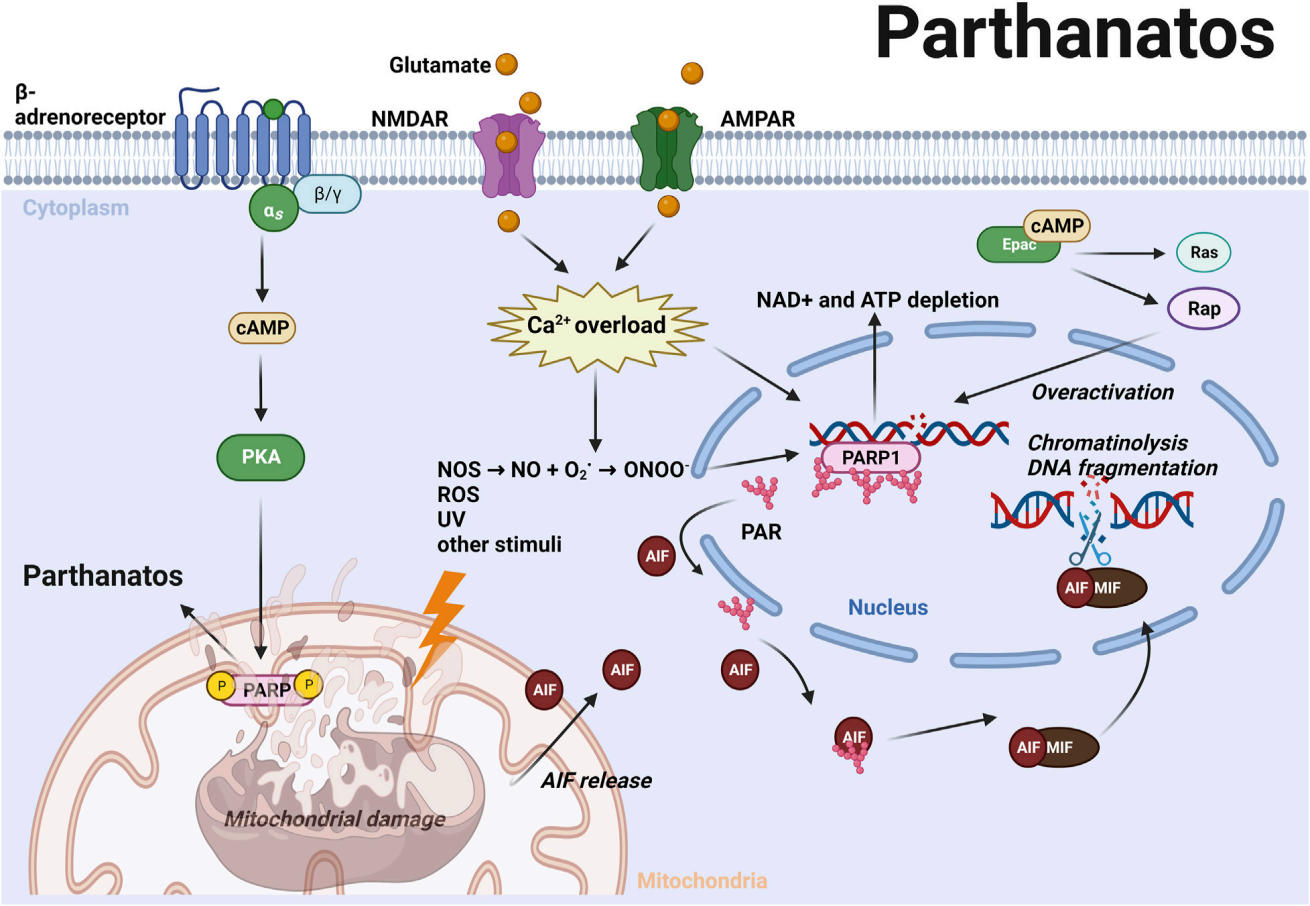
ROS and other stimuli induce DNA damage subsequently leading to the hyperactivation of PARP-1 and PARylation (Andrabi et al., 2008; Narne et al., 2017). Excessive PAR polymers translocate from the nucleus to mitochondria and induce AIF release from mitochondria. AIF recruits MIF into the nucleus and causes chromatinolysis and large-size DNA fragmentation (Wang et al., 2011; Wang et al., 2016). In AD and PD, excessive glutamate activates N-Methyl-D-aspartic acid (NMDA) and α-amino-3-hydroxy-5-methyl-4-isoxazolepropionic acid (AMPA) receptors that induce Ca^2+^ influx and NOS activation, leading to generation of NO and ONOO−, which can potentiate DNA damage and trigger parthanatos (Fatokun et al., 2013). Activation of cAMP/PKA axis promotes phosphorylation of mitochondrial PARP-1, thereby promoting parthanatos (Brunyanszki et al., 2014). Epac activates Rap and H-Ras, and upregulates PARP expression by inhibiting its cleavage (Grandoch et al., 2009b). These mechanisms highlight the role of cAMP signaling in promoting parthanatos via both PKA and Epac. For further details, see text.

Parthanatos is a caspase-independent RCD that is triggered by hyperactivation of poly (ADP-ribose) polymerase (PARP-1) (Galluzzi et al., 2018). Unlike apoptosis, parthanatos occurs without the formation of an apoptotic body or small-size DNA fragmentation (Wang et al., 2009). PARP-1 is vital for repairing single or double strand DNA breaks induced by toxic stimuli such as oxidative stress, nitrosative stress, hypoxia, hypoglycemia, or inflammation (Galluzzi et al., 2018). Severe DNA damage causes PARP-1 overactivation, which subsequently catalyzes a variety of DNA repair proteins via transferring ADP-ribose (PAR) to itself or its target proteins (Andrabi et al., 2008; Narne et al., 2017). However, excessive poly (ADP-ribosyl)ation (PARlyation) causes nicotinamide adenine dinucleotide (NAD^+^) and ATP depletion, which in turn contributes to parthanatotic cell death (Figure 3). Furthermore, the accumulated PAR polymers translocate from the nucleus to mitochondria and induce the release of apoptosis-inducing factor (AIF) from mitochondria. AIF recruits macrophage migration inhibitory factor (MIF) into the nucleus and causes parthanatotic chromatinolysis and large-size DNA fragmentation inside the nucleus (Wang et al., 2011; Wang et al., 2016). In AD and PD, excessive release of the neurotransmitter glutamate activates N-Methyl-D-aspartic acid (NMDA) receptors or α-amino-3-hydroxy-5-methyl-4-isoxazolepropionic acid (AMPA) receptors, further induces Ca^2+^ influx and activation of nitric oxide synthase (NOS) (Fatokun et al., 2013; Gerace et al., 2014). Nitric oxide (NO) reacts with superoxide anion (O_2_^−^) which is a common ROS in neurodegenerative disorders, and forms into peroxynitrite (ONOO^−^). NO and ONOO^−^ can potently induce DNA damage and consequently lead to parthanatotic neuronal death (Fatokun et al., 2013) (Figure 3; Table 1).

**TABLE 1:** Studies of cAMP modulators in *in vitro* models of regulated cell deaths.

Research	Regulated cell deaths	cAMP modulators	Target	*Model*	Main research findings
Brunyanszki et al. (2014)	Parthanatos	Propranolol	β-receptor antagonist	U937	β -adrenoceptor/cAMP/PKA axis phosphorylates PARP-1 subsequently leading to parthanatos
		DDA	AC inhibitor	C2C12	
		PKAi	PKA inhibitor		
		Isoproterenol	β-receptor agonist		
		Forskolin	AC activator		
		8-Br-cAMP	PKA&Epac activator		
Grandoch et al. (2009a)	Parthanatos	8-pCPT-2′-O-Me-cAMP	Epac activator	U937	Epac activation leads to inhibition of PARP cleavage
Grandoch et al. (2009b)	Parthanatos	8-Br-cAMP	PKA&Epac activator	WEHI 231	Epac upregulates PARP expression via Rap1 and H-Ras
		dd-Ado	AC inhibitor		
		SQ22536	AC inhibitor		
		H-89	PKA inhibitor		
		Forskolin	AC activator		
		Rp-8CPT-cAMP	PKA inhibitor		
		8-pCPT-2′-O-MecAMP	Epac activator		
Bokoch et al. (2009)	Netosis	H-89	PKA inhibitor	HEK293	NOXA1 phosphorylation by PKA recruits 14-3-3 protein subsequently inhibiting Nox holoenzyme assembly and potentially preventing netosis
		Forskolin	AC activator	HT-29	
Eby et al. (2014)	Netosis	Forskolin	AC activator pan-PDE inhibitor	Neutrophil	Elevated cAMP by AC inhibits NETs formation and oxidative burst
		IBMX			
Stahl-Meyer et al. (202)	Lysosome dependent cell death	Forskolin	AC activator	MCF7	Lysosphingolipid-induced lysosome-dependent cell death requires cAMP signaling seemingly sensitizing cells to lysosome-dependent cell death
				Fibroblast	
Sung et al. (2020)	Senescence	H-89	PKA inhibitor	Vascular smooth muscle cell	cAMP/PKA-SIRT1 seem to mediate antisenescence mechanisms
		Forskolin	AC activator		
Wang et al. (2022)	Senescence	Forskolin	AC activator	mesenchymal stem cells	cAMP alterations impact senescence and senescence-related inflammatory phenotypes
		SQ22536	AC inhibitor		
Ishii et al. (2019)	Ferroptosis	8-Br-cAMP	PKA&Epac activator	Yeast mitochondria	PKA suppresses mitochondria and indirectly inhibits Nrf2
Musheshe et al. (2022)	Ferroptosis	CE3F4	Epac1 inhibitor	HT-22	Epac1 inhibition reduces ROS and lipid peroxidation subsequently preventing ferroptosis
		ESI-05	Epac2 inhibitor		
		ESI-09	Epac inhibitor		
		8-pCPT-2′-O-Me-cAMP	Epac activator		

AC, adenylyl cyclase; DDA, 2′,3′-dideoxyadenosine; PKA, protein kinase A; epac, exchange protein directly activated by cAMP; IBMX, 3-isobutyl-1-methylxanthine; PARP, Poly (ADP-ribose) polymerase; Nox, NADPH oxidase; NETs, neutrophil extracellular traps; SIRT1, Sirtuin 1; Nrf2, nuclear factor erythroid 2-related factor 2.

Many studies have indicated that parthanatos is associated with AD and PD. In AD, Aβ has been shown to activate PARP-1 in various cell models, including microglia, astrocytes, and neuronal/glial cell cultures (Martire et al., 2013; Wang and Ge, 2020). Love et al. (1999) reported that PARP-1 and PAR were more expressed in *postmortem* AD brains compared to controls. Interestingly, in those AD brains, PAR containing cells were mainly represented by neurons and rarely by astrocytes or microglia, suggesting that parthanatos mainly occurs in neurons in AD (Love et al., 1999). Additionally, Lee et al. (2012) found AIF was significantly elevated in hippocampal CA1 region at early stages of AD and CA2 region at advanced stages of AD. In PD, a recent study reported that parthanatos was triggered by α-synuclein pre-formed fibrils (α-syn PFFs) via NOS mediated DNA damage, while other RCDs including necroptosis and autophagy were not involved in α-synuclein induced cell death, which may imply that parthanatos is a prominent RCD in PD pathology. Furthermore, PAR promoted the fibrillization of α-synuclein into a new strain with 25-fold greater toxicity, which further activated PARP-1. The level of PAR was found to be increased in the cerebrospinal fluid (CSF) and substantia nigra of PD patients, further confirming that PARP-1 activation is involved in PD pathogenesis (Kam et al., 2018) (Table 1).

Several PARP inhibitors have been developed and approved for clinical trials of cancer treatment, but their precise effects on AD and PD still need to be determined (Zhou et al., 2021). On one hand, inhibition of PARP-1 prevents parthanatos via mitigating over-active PARP-1. For example, it was found that treatment with the PARP-1 specific inhibitor 3AB prevented ROS-mediated cell death parthanatos and downregulated parthanatotic pathway in human SH-SY5Y neuroblastoma cells (Wang et al., 2018). On the other hand, PARP-1 depletion also leads to dysfunctional DNA repair machinery, severe DNA damage, and consequently promoting cell death, which has been used as the strategy in cancer treatment (Zhou et al., 2021). Therefore, considering the unknown side effect of long-term PARP-1 inhibition, the use of PARP-1 inhibitors in treating long-term AD and PD should be applied with caution. Alternative approaches to prevent parthanatos include targeting AIF and MIF. Inhibition of AIF release from mitochondria, its interaction with binding proteins such as cyclosporin A, or its translocation into the nucleus with compounds such as N-phenylmaleimide can prevent AIF-mediated parthanatos (Fatokun et al., 2013). Similarly, adenoviral knockdown of MIF in a rat spinal cord model has been found to reduce oxidative stress-induced parthanatos in neurons (Yang et al., 2020), and MIF antagonists such as antibodies, inhibitors, and fragment peptides of MIF have been successfully used to diminish parthanatos driven cell death (Song et al., 2022). Recent studies have shown promising results using the MIF nuclease inhibitor PAANIB-1 *in vivo* to mitigate α-synuclein or 1-methyl-4-phenyl-1,2,3,6-tetrahydropyridine (MPTP)-induced neurodegeneration (Park et al., 2022). These studies offer potential usage of parthanatos associated therapeutics in the future treatment of AD and PD.

Intriguingly, it was demonstrated that activation of the cAMP/PKA axis promotes mitochondrial PARP-1 activation induced by ROS (Brunyanszki et al., 2014) (Figure 3). Stimulation of the Gs-coupled β-adrenoceptor increase the cellular cAMP levels subsequently leading to the activation of PKA. Activated PKA translocates to the mitochondria where it modulates parthanatos by phosphorylating PARP-1 (Brunyanszki et al., 2014). The activation of Epac has also been found to potentially contribute to parthanatotic cell death by inhibiting the cleavage of PARP (Grandoch et al., 2009a) (Figure 3). Additionally, their findings suggest that Epac upregulates PARP expression via Rap1 and H-Ras, as inhibition of these proteins has been observed to promote PARP cleavage (Grandoch et al., 2009b) (Table 1). Taken together modulation of parthanatos driven cell death might rely on the cAMP effectors PKA and Epac, and modulation of these cAMP signaling pathways might bear the potential for future treatment options in AD and PD.

### Netosis

**FIGURE 4:**
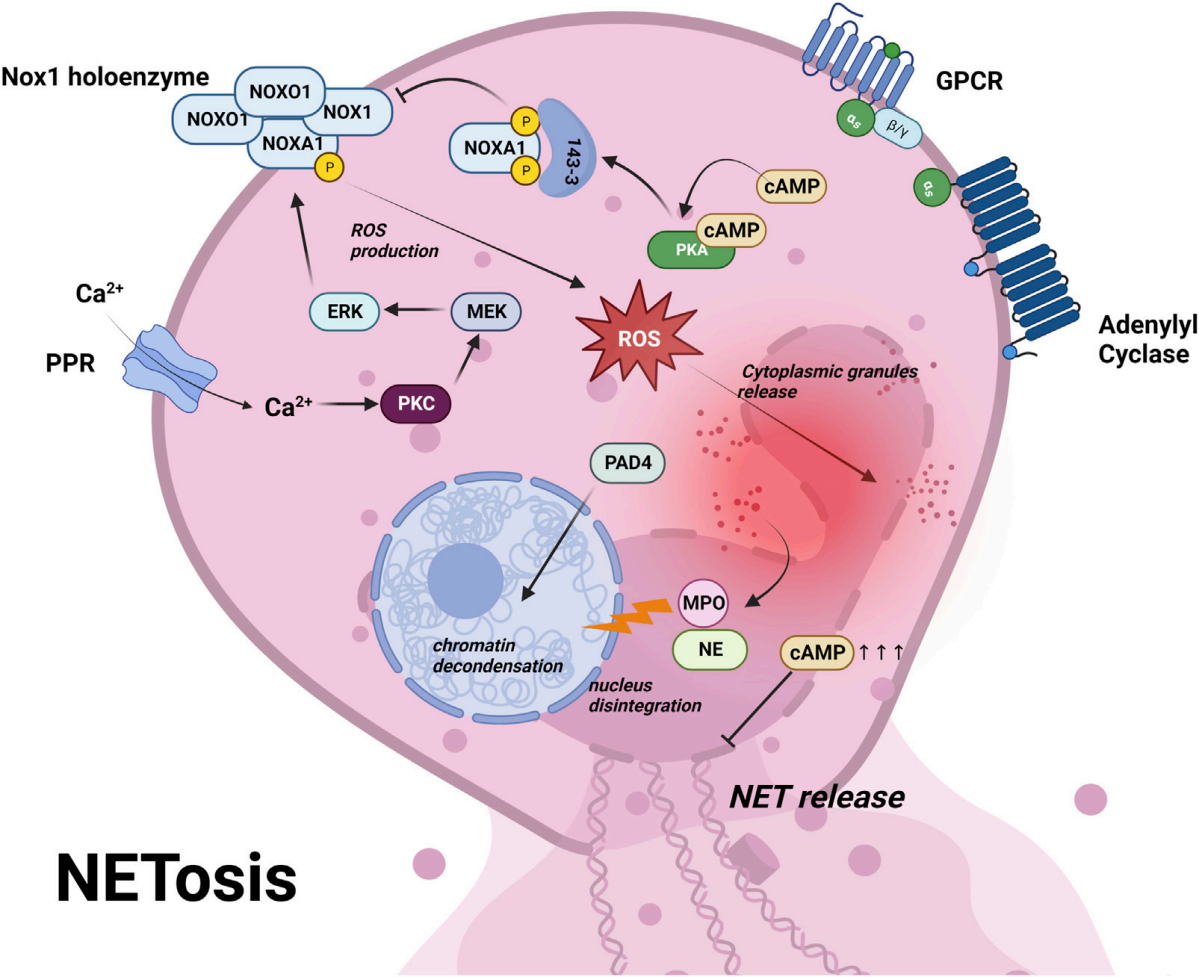
Netosis is initiated by activation of neutrophils via pattern recognition receptors (PRRs) and a subsequent influx of Ca^2+^ (Huang et al., 2022). This triggers a cascade of events involving Ca^2+^-dependent PKC, Raf-MEK-ERK pathway, NAPDH oxidase (NOX) phosphorylation, leading to ROS production (Hakkim et al., 2011; Dahlgren et al., 2019) (panel 4). The excessive ROS causes cytoplasmic granule degradation and release of elastase (NE) and myeloperoxidase (MPO) to the nucleus, along with peptidyl arginine deiminase 4 (PAD4), leading to chromatin decondensation and ultimately resulting in cell rupture and NETs release (Papayannopoulos et al., 2010) (panel 4). PKA phosphorylation of NOXA1 recruits 14-3-3 proteins, which block the assembly of NOX1 holoenzyme, ultimately preventing ROS production (Bokoch et al., 2009). Moreover, supraphysiological cAMP inhibits the formation of NETs and ROS burst (Eby et al., 2014). These findings highlight the role of cAMP signaling in inhibiting netosis via PKA. For further details, see text.

Netosis is a type of RCD dependent on neutrophil extracellular traps (NETs) in response to infection or injury (Galluzzi et al., 2018). Activated neutrophils undergo netosis and release NETs to limit the growth or kill various pathogenic microorganisms including bacteria, fungi, virus and parasite (Mutua and Gershwin, 2021). Netosis is initiated by activation of neutrophils via a variety of pattern recognition receptors (PPRs) including toll-like receptors, NOD-like receptors and C-type lectin receptors, followed by Ca^2+^ influx (Huang et al., 2022). This elevated Ca^2+^ triggers the activation of a subset of calcium-dependent protein kinase C (PKCs) and downstream Raf-MEK-ERK cascades, which phosphorylate NADPH oxidase (NOX) and enhance ROS production (Hakkim et al., 2011; Dahlgren et al., 2019) (Figure 4). Excessive ROS leads to the degradation of cytoplasmic granule and causes the release of elastase (NE) and myeloperoxidase (MPO) into the nucleus, together with peptidyl arginine deiminase 4 (PAD4), leads to chromatin decondensation, ultimately resulting in cell rupture and NETs release (Papayannopoulos et al., 2010) (Figure 4; Table 1).

The blood-brain barrier (BBB) is a highly semipermeable border that insulates brain parenchyma from circulating leukocytes. Neutrophils infiltrated the BBB and migrated into parenchyma via adhesion to blood vessels, and released NETs and IL-17 in transgenic AD models (Zenaro et al., 2015). Furthermore, AD pathology and cognitive deficits were attenuated after temporary depletion of neutrophils or inhibition of neutrophil trafficking (Zenaro et al., 2015). In the same study, neutrophils and NETs were also found to accumulate around Aβ plagues in AD patients, while neutrophils from age-matched controls were remained in blood vessels. In a recent study, an increased adhesion of neutrophils to small blood vessels, along with NETs, were observed in an AD mouse model and AD patients, suggesting that netosis contributes to BBB breakdown in AD (Smyth et al., 2022). These available data suggest that netosis is involved in the pathogenesis of AD.

Inhibition of NET formation has already been used as a therapeutic approach against autoimmune and inflammatory diseases. NOX is a good target in netotic pathway and has entered several preclinical/clinical trials (Chamardani and Amiritavassoli, 2022). NOX inhibitors such as Apocynin and VAS2870, prevented ROS and netosis in human neutrophils (Vorobjeva et al., 2020). In addition to the regulation of NOX activity through the PKC pathway, NOX activity can also be regulated by PKA, which phosphorylates on NOXA1 and recruits regulatory14-3-3 proteins. The binding of 14-3-3 blocks the assembly of NOX1 holoenzyme, consequently preventing ROS production (Bokoch et al., 2009). In addition, it was reported that cAMP inhibited the formation of NETs and ROS burst (Eby et al., 2014) (Figure 4; Table 1). These studies suggest that activation of cAMP/PKA pathway may prevent netosis via NOX inhibition. Until now studies with focus on Epac and a potential link to PD are still missing and might be worthwhile to pursue in the future.

### Lysosome-dependent cell death

**FIGURE 5:**
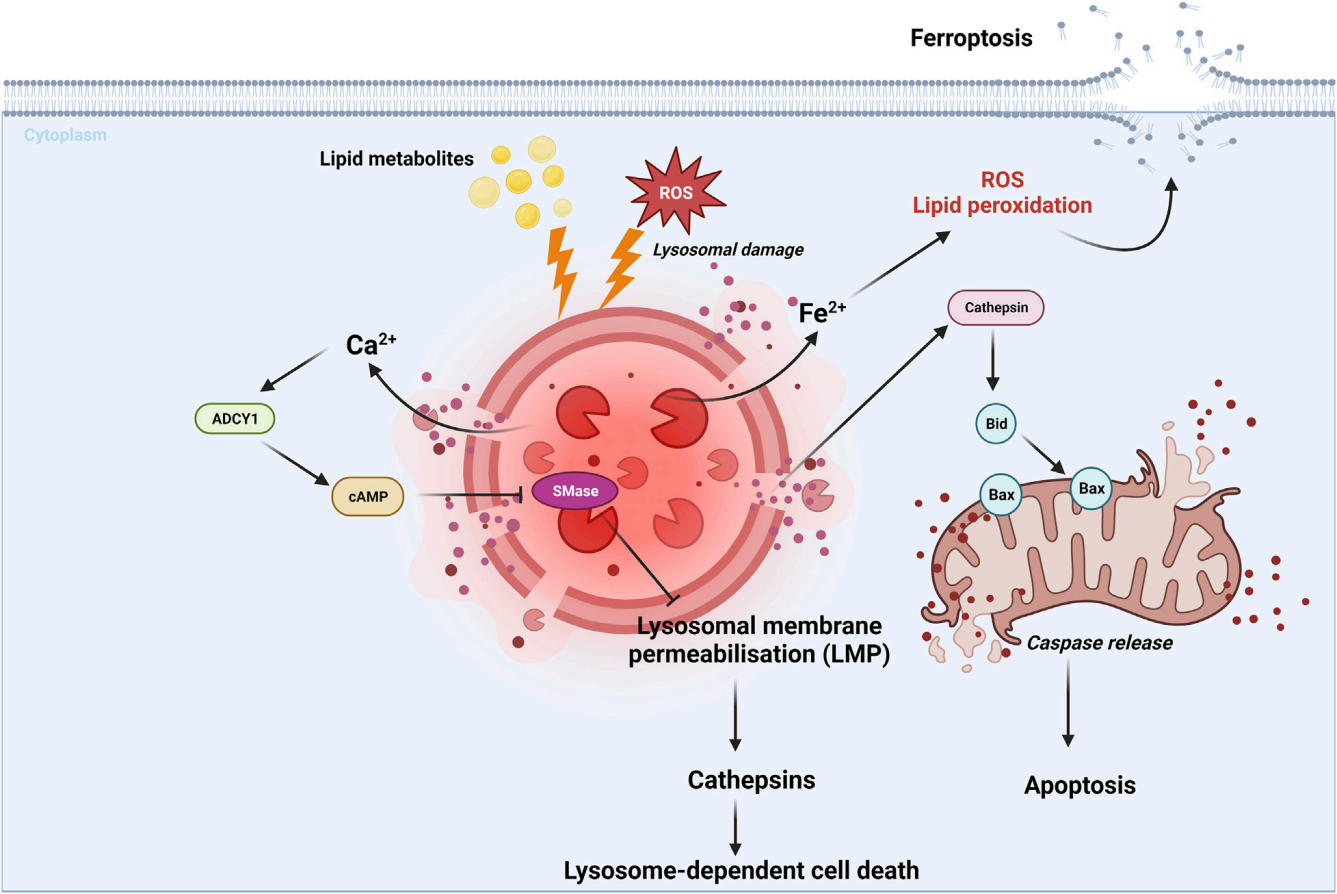
Lysosome-dependent cell death is triggered by ROS or other stimuli, leading to LMP and a release of cathepsins into cytosol. Cathepsins catalyze multiple substrates, including Bid and anti-apoptotic proteins and initiate caspase-dependent cell death (Taniguchi et al., 2015; Gómez-Sintes et al., 2016). Additionally, lysosome-dependent cell death can activate other RCDs, such as apoptosis and ferroptosis, through the leakage of Bid-specific cathepsins and iron-rich content, respectively (Kurz et al., 2008; Gao et al., 2018). Lysosphingolipid induced lysosomal cell death in a process involving Ca^2+^-dependent adenylyl cyclase 1 (ADCY1), followed by an cAMP elevation, and finally inhibition of lysosomal acid sphingomyelinase (SMase) (Anand et al., 2019; Stahl-Meyer et al., 2022). These findings highlight the role of cAMP signaling in promoting lysosome-dependent cell death via ADCY1. For further details, see text.

Lysosome-dependent cell death is a type of RCD mediated by the lysosomal release of the proteolytic enzyme cathepsin. This cell death is induced by various stimuli including ROS, lysosomotropic detergents, dipeptide methyl esters and lipid metabolites (Tang et al., 2019) (Figure 5). After lysosomal membrane permeabilization (LMP), cytosolic cathepsins catalyze multiple substrates, such as Bid and anti-apoptotic proteins, and subsequently initiate caspase-dependent cell death (Taniguchi et al., 2015; Gómez-Sintes et al., 2016). It is worth noting that lysosome-dependent cell death can also trigger other forms of RCD, such as apoptosis and ferroptosis, which are respectively caused by the lysosomal leakage of Bid-specific cathepsins and iron-rich content (Kurz et al., 2008; Gao et al., 2018) (Figure 5; Table 1).

It was reported that oligomeric Aβ42 induced LMP and subsequently released lysosomal enzymes, including cathepsins, resulting in neuronal death (Hook et al., 2020). In AD patients, cathepsin D and other lysosomal proteins were increased in blood exosome up to 10 years before clinical symptoms (Goetzl et al., 2015). Furthermore, active cathepsin D and cathepsin B were elevated and co-localized with senile plaques in the AD brains (Cataldo and Nixon, 1990). This can be attributed to the role of these cathepsins in Aβ degradation (Cermak et al., 2016). In a study of PD patients and a PD mouse model, reduced lysosomal proteins was found to be associated with increased Lewy body in nigral (Vila et al., 2011). LMP was also confirmed in 1-methyl-4-phenylpyridinium (MPP^+^)-induced PD cell model (Vila et al., 2011). These studies suggest that lysosome-dependent cell death is involved in pathogenesis of AD and PD.

A recent study found that lysosphingolipids induced lysosome-dependent cell death required activation of cAMP signaling pathways (Stahl-Meyer et al., 2022). Furthermore, elevated cAMP induced by AC activator forskolin, was found to sensitize cells to lysosomal cell death when co-treated with low concentrations of lysosphingolipids (Stahl-Meyer et al., 2022) (Table 1). This lysosomal cell death pathway probably started with a fast release of Ca^2+^ from lysosomes, followed by activation of Ca^2+^-dependent adenylyl cyclase 1 (ADCY1), which ultimately inhibited lysosomal acid sphingomyelinase (SMase) (Anand et al., 2019; Stahl-Meyer et al., 2022) (Figure 5; Table 1). However, it is important to note that further studies are required to fully understand how cAMP pathway is involved in lysosome-dependent cell death.

### Cellular senescence

**FIGURE 6:**
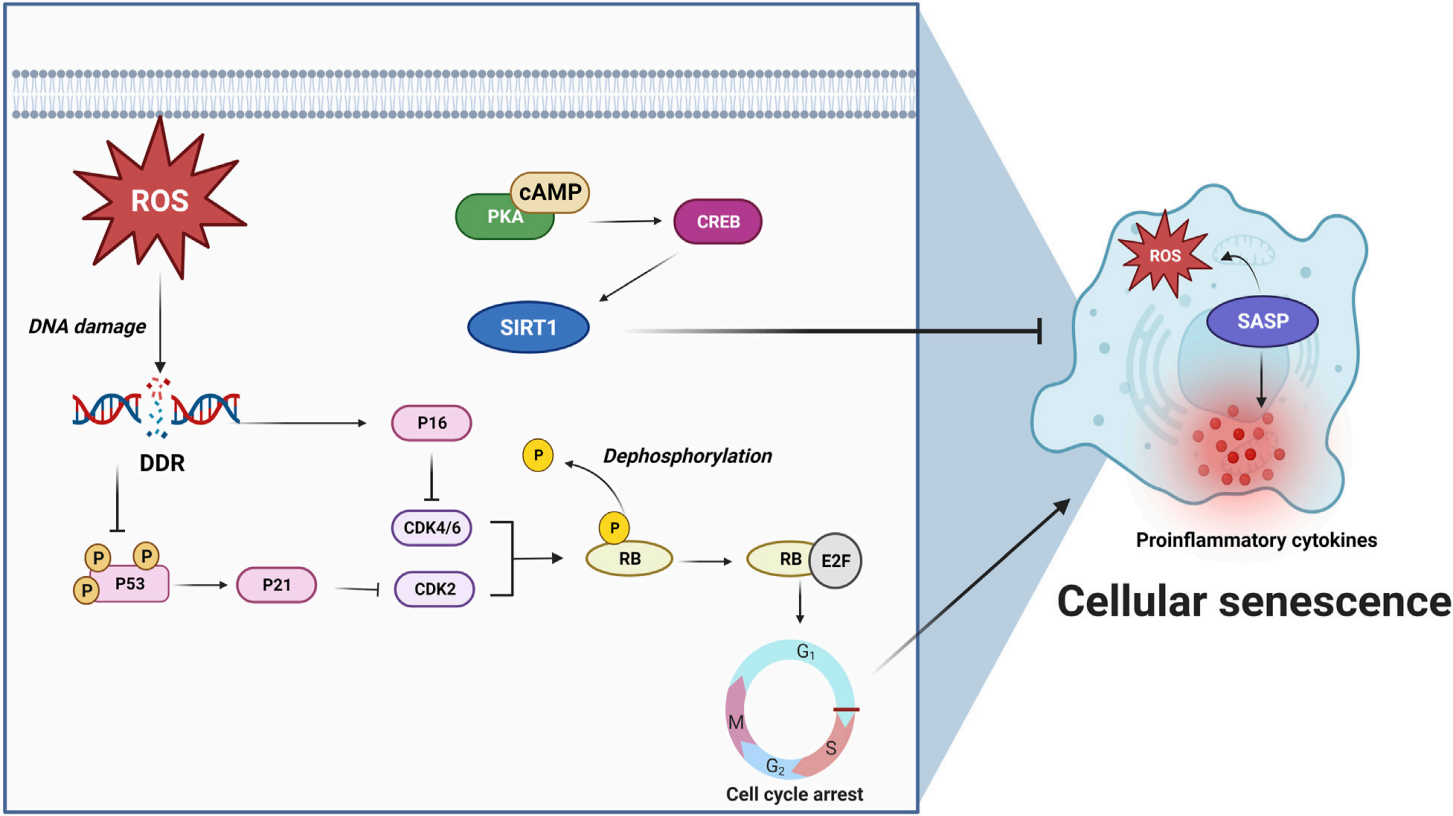
ROS or other stressors initiates cellular senescence via DNA damage and subsequent DNA damage response (DDR) activation. DDR results in p53 phosphorylation (Ramirez et al., 2001; Turenne et al., 2001; Nakamura et al., 2008) and p21 activation, leading to CDK2 inhibition and Rb/E2F-mediated G1 to S cell cycle arrest (Krenning et al., 2014). In addition, p16 can specifically inhibit CDK4/6 and maintain senescent cell cycle arrest (Stein et al., 1999; Ruas et al., 2007). Senescent cells produce proinflammatory factors and ROS when they turn into SASPs. cAMP/PKA/CREB pathway activates anti-senescence protein, Sirtuin 1 (SIRT1), and prevents cellular senescence (Sung et al., 2020; Zhu et al., 2022). These findings highlight the role of cAMP/PKA signaling in inhibiting cellular senescence via activating SIRT1. For further details, see text.

Cellular senescence refers to a pathophysiologial process that normal cells lose their proliferation abilities and undergo irreversible arrest in cell cycle when they reach their limits of replicative lifespan (Galluzzi et al., 2018). Cellular senescence was previously thought to be a type of RCD being induced by telomere shortening and other stressors, such as ROS, mitochondrial damage and tumor suppressor proteins (Campisi, 2013). ROS and other stressors can damage DNA and subsequently trigger DNA damage response (DDR) to phosphorylate a cell cycle repressor, p53 (Ramirez et al., 2001; Turenne et al., 2001; Nakamura et al., 2008). p53 further activates cyclin-dependent kinase 2 (CDK2) inhibitor p21 and results in decreased phosphorylation of retinoblastoma protein (Rb), which binds and inactivates transcription factor E2F, and subsequently arrests the cell cycle from G1 to S (Krenning et al., 2014). Additionally, p16^INK4a^ can specifically inhibit CDK4 and CDK6 and thereby maintain the senescent suspension of cell cycle (Stein et al., 1999; Ruas et al., 2007). Senescent cells can release proinflammatory factors and ROS when they are turned into senescence-associated secretory phenotypes (SASPs) (Coppé et al., 2010). p38 mitogen-activated protein kinases (p38MAPK), nuclear factor kappa-light-chain-enhancer of activated B cells (NF-κB), cyclic GMP-AMP synthase (cGAS) and mammalian target of rapamycin (mTOR) are implicated in the SASP conversion process (Kritsilis et al., 2018) (Figure 6; Table 1).

Cellular senescence was observed in wide range of cell types in central nervous system, including neurons, astrocytes, microglia, oligodendrocytes, oligodendrocyte progenitor cells and neural stem cells (NSCs) (Kritsilis et al., 2018). Senescent neurons and glia cells were found to be linked with AD and PD (Turnquist et al., 2016; Bussian et al., 2018; Chinta et al., 2018; Kritsilis et al., 2018), and pathological hallmarks of AD and PD, such as Aβ, tau, and α-synuclein, were reported to induce senescence in *in vitro* or *in vivo* models (Verma et al., 2021; Liu, 2022). In AD patients and mouse models, elevated senescent markers, p16^INK4a^, p21^CIP1^, p53 and senescence associated β-Galactosidase (SA-β-Gal) were observed (Arendt et al., 1996; Arendt et al., 1998; McShea et al., 1997; Lüth et al., 2000; Engidawork et al., 2001; Hochstrasser et al., 2011; Esteras et al., 2012; Esteras et al., 2013; Bhat et al., 2012; Campisi, 2013; Yates et al., 2015). Similarly, p16^INK4a^ and SA-β-Gal were increased in PD patients (van Dijk et al., 2013; Chinta et al., 2018). Studies revealed that SASP factors, including interleukin-1β (IL-1β), interleukin-6 (IL-6), transforming growth factor-β (TGF-β), and tumor necrosis factor-α (TNF-α), were upregulated in AD, as evidenced by their elevation in the brains, CSF, and serum of AD patients (Blum-Degena et al., 1995; Dursun et al., 2015; Huell et al., 1995; Lai et al., 2017; Rea et al., 2018; Swardfager et al., 2010; Wood et al., 1993). Similarly, increased levels of IL-1β, IL-6, and TNF-α were observed in the striatum and CSF of PD patients (Blum-Degena et al., 1995; Hofmann et al., 2009; Scalzo et al., 2010; Lindqvist et al., 2012; Dursun et al., 2015; Chinta et al., 2018). Furthermore, hippocampal p38MAPK was reported to be activated during the early stage of AD (Sun et al., 2003). Taken together, these findings suggest that cellular senescence is very likely involved in the pathogenesis of AD and PD.

Cellular senescence has emerged as a potential target for the treatment of AD and PD. Senolytic treatment, which selectively induces death of senescent cells, has shown promise as a novel therapeutic strategy. However, it is still controversial which cell type should be the primary target of senolysis. A recent study found that oligodendrocyte progenitor cell (OPC) was the only glia cell type showing elevated senescent markers in AD patients and an Aβ mouse model. Selective elimination of senescent OPCs improved cognitive function, reduced neuroinflammation, and decreased Aβ load (Zhang et al., 2019). Another study correlated astrocytes and microglia with increased p16^INK4a^ in P19 tau pathology mice, and demonstrated that genetic and pharmacological clearance of those cells could reduce gliosis, neurofibrillary tangles (NFTs), neuronal loss and cognitive impairment (Bussian et al., 2018). In contrast, the third study suggested that NFT-containing neurons were the primary target of senolysis in human and mouse AD brains. Treatment with senolytics reduced NFT density, neuronal loss and ventricular atrophy in a tau transgenic mouse model (Musi et al., 2018). Additionally, a PD study showed that senolytic treatment could reduce neuropathology and improve motor deficits (Chinta et al., 2018). Taken together, these studies provide compelling evidence for the potential of senolysis as a therapeutic approach for AD and PD. However, further research is needed to clarify the optimal cell types to target and the most effective senolytic treatment strategies.

Sirtuin 1 (SIRT1) is commonly accepted as an anti-senescence protein, as it possesses deacetylase activity that can be activated through cAMP signaling (Aslam and Ladilov, 2022). Sung et al. (2020) recently demonstrated that overexpression or activation of SIRT1 attenuated senescence via cAMP/PKA pathway in vascular smooth muscle cells. Another study revealed that senescence-accelerated mouse prone 8 (SAMP8) mice, which showed neurodegeneration induced by Aβ and accelerated senescence, expressed lower levels of cAMP, phosphorylated PKA, and phosphorylated CREB (Hou et al., 2022). Similarly, in a rat epithelial cell line, interleukin-13 (IL-13) induced senescence downregulated phosphorylated CREB (Zhu et al., 2022) (Figure 6). These studies suggest that activation of cAMP/PKA/CREB pathway prevents cellular senescence. However, in contrast to these findings, Wang et al. (2022) found that elevated cAMP induced by forskolin triggered cellular senescence in human-derived mesenchymal stem cells, while inhibition of cAMP by SQ22536 prevented against forskolin-induced senescence and senescence-related inflammatory phenotypes. These studies reveal differential roles of cAMP signaling in cellular senescence across various cells and tissues. Therefore, further research is necessary to fully understand the role of cAMP signaling in senescence and its potential therapeutic implications.

### Ferroptosis

Dysregulation of trace metals is one of the pathological features in AD and PD. In AD, zinc, copper and iron accumulate in senile plaques, with zinc and copper inducing a conformational change in Aβ and resulting in non-fibrillar Aβ oligomerization, while iron is likely to be associated with plaques in a ferritin-bound form (Grundke-Iqbal et al., 1990; Lovell et al., 1998; Tiiman et al., 2013). Similarly, in PD, iron accumulation in Lewy bodies has been observed and implicated in accelerating α-synuclein aggregation (Hashimoto et al., 1999; Castellani et al., 2000; Uversky et al., 2001). Iron overload has been detected in specific brain regions including the parietal cortex, motor cortex, and hippocampus in AD patients (Bartzokis et al., 1994; Bartzokis and Tishler, 2000; Pfefferbaum et al., 2009; Langkammer et al., 2014; Tao et al., 2014), while increased iron levels have been observed in the substantia nigra in PD patients (Dexter et al., 1987). Furthermore, increased ferritin level in CSF is linked with the progression of AD (Ayton et al., 2018; 2015). The presence of reactive redox ions acts as catalysts to generate ROS via Fenton and Haber-Weiss reactions and further initiate lipid peroxidation, which have been detected in AD and PD patients (Tsang and Chung, 2009; Butterfield et al., 2010; Bradley-Whitman and Lovell, 2015). In addition, lipid peroxidation also causes breakdown of blood brain barrier and, therefore, exacerbates progression of early AD and PD (Chen et al., 2022).

**FIGURE 7:**
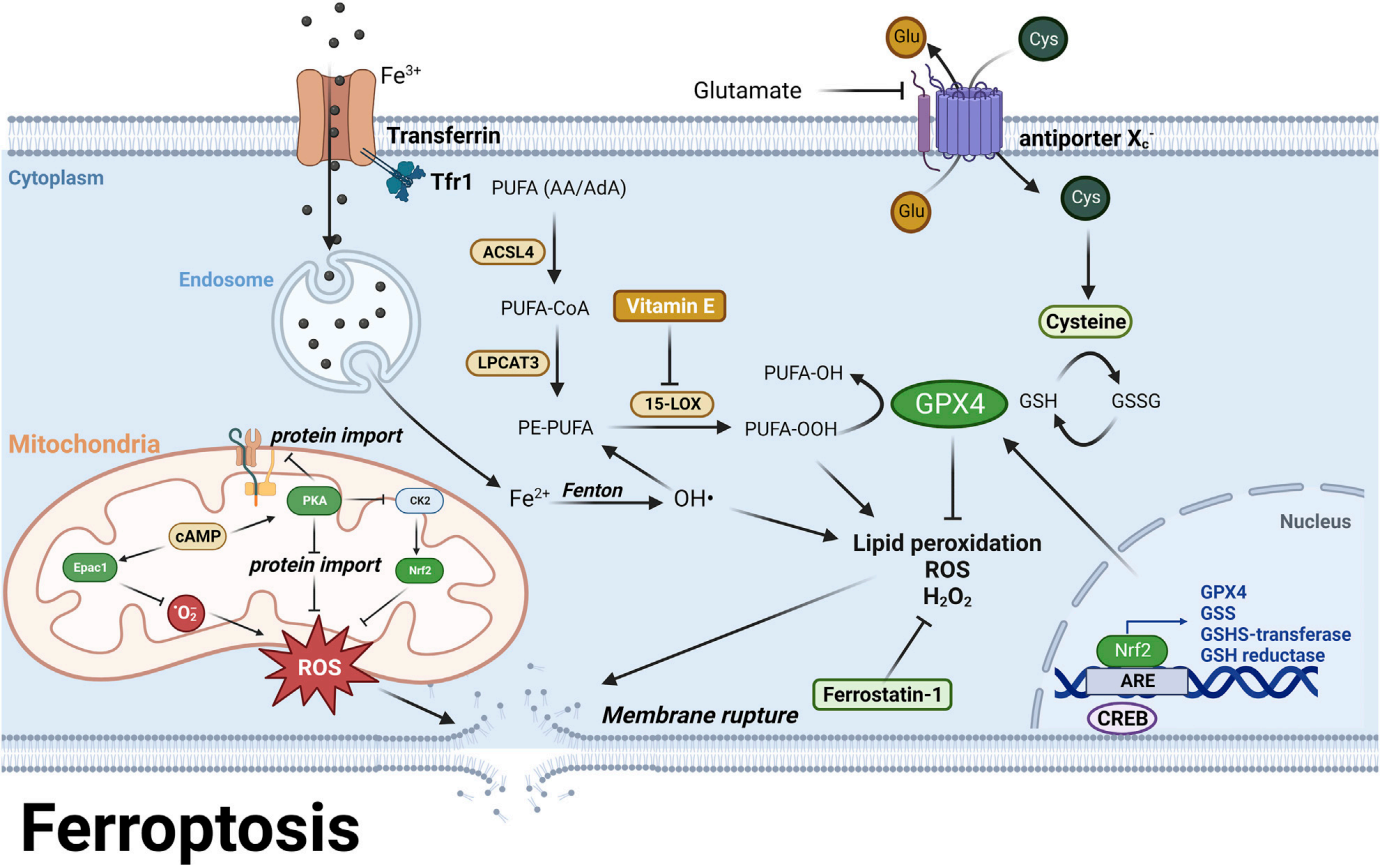
PUFA are stepwise oxidized into lipid hydroperoxides, PUFA-OOH by ACSL4, LPCAT3, and 15-LOX (Kagan et al., 2017; Li and Li, 2020). GPX4 uses GSH as a substrate to catalyze lipid hydroperoxides into hydroxy derivatives, limiting lipid peroxidation (Lewerenz et al., 2013). Excessive glutamate or System Xc-inhibition causes GSH depletion and attenuates GPX4 activity, leading to lipid peroxidation and ferroptosis (Bannai and Kitamura, 1980). Moreover, iron overload generates hydroxyl radicals via Fenton reaction, which also contributes to lipid peroxidation (Yang et al., 2016). Epac1 inhibition reduces ROS levels and lipid peroxidation by decreasing mitochondrial superoxide (Musheshe et al., 2022), while PKA suppresses mitochondrial activation and ROS production via inhibiting protein import (Ishii et al., 2019). In the meantime, PKA indirectly inhibits Nrf2 via outcompeting with Nrf2 regulator CK2. In addition, CREB regulates GPX4 transcription to prevent ferroptosis (Wang et al., 2021). These findings highlight the role of cAMP signaling in preventing ferroptosis via both PKA and mitochondrial Epac. For further details, see text.

Ferroptosis is a type of RCD that is characterized by severe lipid peroxidation due to ROS generation and iron overload, both of which are also the pathological features in AD and PD (Galluzzi et al., 2018) (Figure 7). The lipid hydroperoxides are originated from polyunsaturated fatty acids (PUFA), such as arachidonoyl (AA) and adrenoyl (AdA) phospholipids, which are stepwise oxidized into PUFA-OOHs by long-chain-fatty-acid-CoA ligase 4 (ACSL4), lysophosphatidylcholine acyltransferase 3 (LPCAT3) and 15-lipoxygenase (15-LOX), and eventually act as ferroptotic signals and execute ferroptosis (Kagan et al., 2017; Li and Li, 2020). Glutathione peroxidase 4 (GPX4) is the only enzyme that catalyzes lipid hydroperoxides into hydroxy derivatives within biological membranes (Conrad and Friedmann Angeli, 2017). GPX4 oxidizes its substrate glutathione (GSH) into glutathione disulfide (GSSG) to clear lipid hydroperoxides (Li and Li, 2020). GSH is synthesized from a nonessential amino acid cysteine, which limits the rate of GSH synthesis (Lewerenz et al., 2013). Cysteine is directly transported into cells or in its oxidative form, cystine, via the cystine/glutamate antiporter System X_c_^−^ (Lewerenz et al., 2013). Inhibition of System X_c_^−^ by its inhibitors or high concentration of glutamate results in decreased intracellular cysteine pool and further leads to GSH depletion (Bannai and Kitamura, 1980). GSH depletion attenuates the activity of GPX4 and activates 15-LOX, and further trigger lipid peroxidation and ferroptosis (Lewerenz et al., 2018). Iron overload also contributes to lipid peroxidation since ferrous ion is a cofactor of 15-LOX (Yang et al., 2016). Additionally, the major metabolite of 15-LOX, 15-hydroxyeicosatetraenoic acid (15-HETE), disrupts the tight junctions of endothelial cells in blood brain barrier and results in BBB dysfunction (Chattopadhyay et al., 2014) (Figure 7).

It has been demonstrated a correlation between the cognitive decline in AD and PD and the reduction of GSH in hippocampus and frontal cortex (Mandal et al., 2015). Iron and copper have been found to promote GSH loss and cell death in HT22 hippocampal neurons (Maher, 2018), and decreased GSH levels have been observed in dopaminergic neurons in PD brains (Pearce et al., 1997). A conditional ablation of GPX4 in mouse forebrain neurons exhibited AD-like dysfunctions in learning and memory, as well as neurodegeneration in the hippocampus (Hambright et al., 2017). A recent RNA-seq analysis of AD patients reported that ferroptosis related genes, such as GPX4 and glutathione synthase (GSS), were downregulated at early AD and upregulated at late AD, suggesting that neuronal cells gradually compensate ferroptotic cell death by increasing antioxidant proteins with the progression of AD (Majerníková et al., 2021) (Figure 7). Interestingly, neurons expressed more ferroptosis related genes than glia cells in AD patients, probably revealing that ferroptosis initially affects neurons and subsequently spread to glia cells (Majerníková et al., 2021). This could be due to the fact that glial cells produce and transfer antioxidants to neurons, while neurons express antioxidant genes to a lesser extent (Ishii et al., 2019) (Table 1). These studies highlight the role of ferroptosis as a major type of RCD in AD and PD.

Inhibition of ferroptosis could be a strategy to treat AD and PD. The major anti-ferroptosis drugs are divided into three categories, 1) iron chelators, 2) antioxidants, 3) modulators of GSH synthesis (Lane et al., 2018; Majerníková et al., 2021). Examples of iron chelators include deferoxamine (DFO) and clioquinol, which have shown promise in delaying AD and PD progression in preclinical models (Grossi et al., 2009; Lei et al., 2012; Sripetchwandee et al., 2016; Finkelstein et al., 2017). However, those iron chelators still face limitations before clinical trials, including inability to specifically target at iron-overloaded brain regions and inconsistent dose regimen across different clinical trials (Derry et al., 2020; Farr and Xiong, 2021). Antioxidants attenuate ROS production via either trapping free radicals or inhibiting 15-LOX (Mabalirajan et al., 2009; Kagan et al., 2017; Zilka et al., 2017). Ferrostatin-1 and vitamin E are two examples of antioxidants that have been studied in the context of AD and PD. Ferrostatin-1 traps radicals and has been shown to decrease neurodegeneration in GPX4 knockout mice (Hambright et al., 2017), while vitamin E inhibits LOX and reduces lipid peroxidation (Mabalirajan et al., 2009; Ju et al., 2021) (Figure 7). A study of GPX4 ablation mice reported that Vitamin E deficient diets accelerated hippocampal degeneration and behavioral dysfunction (Hambright et al., 2017). Clinical trials also reported that vitamin E delayed AD progression by 6 months in mild to moderate AD patients and improve symptoms and GSH levels in PD patients (Epperly et al., 2017; Taghizadeh et al., 2017). However, there is still no consensus on the effectiveness of vitamin E, as another study reported that vitamin E non-respondent patients, showed sharp cognitive decline compared with placebo group (Lloret et al., 2009). Modulation of GSH synthesis related proteins, such as GPX4, is another potential therapy against ferroptosis. It was reported that α-Lipoic acid (LA) upregulated GPX4 expression and thereby reduced lipid peroxidation in a Tau mouse model (Zhang et al., 2018). In addition, the transcription of GPX4 and other antioxidant response element (ARE)-containing genes such as GSH S-transferase, GSH reductase and GSS are regulated by a redox induced transcription factor, nuclear factor erythroid 2-related factor 2 (Nrf2) (Song and Long, 2020). Activation of Nrf2 by its activator, sulforaphane, results in increased GSH level, decreased ROS production and improving memory deficits in various preclinical AD models (Kim, 2021) (Figure 7). Overall, these various approaches to inhibiting ferroptosis offer potential methods for treating AD and PD.

In astrocytes, Nrf2 upregulates antioxidant proteins during mitochondrial activation, while PKA suppresses mitochondrial activation and ROS production via blocking protein import into mitochondria, and indirectly inhibits Nrf2 via outcompeting with Nrf2 regulator, casein kinase 2 (CK2) (Ishii et al., 2019). Besides, it was reported that CREB mitigated ferroptosis via regulating GPX4 transcription in a lung cell line (Wang et al., 2021). In a recent study it was demonstrated that inhibition of Epac1 in the immortalized hippocampal (HT22) neuronal cells led to a reduction in ROS levels and lipid peroxidation by decreasing mitochondrial superoxide levels and subsequently preventing ferroptosis (Musheshe et al., 2022) (Figure 7; Table 1). These studies collectively suggest that the cAMP pathway may suppress mitochondrial activity and thereby prevent ferroptosis and shed light on the potential therapeutic targets for preventing ferroptosis by regulating the cAMP pathway.

## Conclusion and outlook

Both AD and PD severely impact life expectancy and quality of life of millions of people worldwide. Unfortunate, despite the global burden, the search after novel drugs able to diminish or even prevent the pathophysiological disease patterns is still characterized by a very limited progress in the last decades. Intriguingly, recent evidence indicates that cAMP-linked drugs - already approved by both FDA (Food and Drug Administration, U.S.) and EMA (European Medicines Agency, Europe)–such as β-adrenoceptor agonists (e.g., salbutamol, albuterol) and antagonists (e.g., propranolol, sotalol) seem to enjoy some clinical success (Cepeda et al., 2019; Crotty and Schwarzschild, 2022; Fella et al., 2022; Lu’O’Ng and Nguyen, 2013; Mittal et al., 2014; Ramos et al., 2008; Wang et al., 2009; Wang et al., 2011; Andersson et al., 2013; Bond et al., 2007). However, patient benefit, potential underlying mechanisms and prevalence of unwanted treatment outcomes are still a matter of debate.

Global elevators and/or inhibitors of cAMP will have numerous (side) effects based on the widespread expression of the effectors of the cAMP signaling pathway in many cells and tissues including the brain. Therapeutics that specifically activate and/or inhibit effectors of cAMP signaling will likely enjoy greater clinical benefits with fewer side effects. In addition, gaining increasing knowledge about the impact of cAMP effectors distinct from β-adrenoceptors may open new avenues for combination therapies–allowing reduction in the concentration of each active drug component thereby most likely limiting unwanted treatment effects. Such combination therapies are already operational to treat chronic lung disorders (e.g., asthma and chronic obstructive pulmonary disease) and cancer. Neurodegenerative disorders may alter also cAMP effector expression and/or localization, causing dysfunctioning of cAMP signaling. Such mechanisms may profoundly contribute to oxidative stress triggered-RCD mechanisms known to result in neuronal death in AD and PD. Therefore, more research into how specific cAMP signaling play key roles in distinct subcellular compartments is needed.

For this purpose, we provided in this review a comprehensive overview about mechanisms underlying ROS-related novel cell death mechanisms in AD and PD. Notably, cAMP signaling appears to play differential roles in these RCDs. Signaling of cAMP via PKA and Epac promotes parthanatos and induces lysosomal cell death, while signaling of cAMP via PKA inhibits netosis and cellular senescence. Additionally, PKA protects against ferroptosis, whereas Epac1 promotes ferroptosis. Specifically, in parthanatos, PKA and Epac have been implicated in promoting cell death through phosphorylation of PARP-1 and inhibition of PARP cleavage, respectively (Grandoch et al., 2009a; Brunyanszki et al., 2014). In lysosomal cell death, elevated cAMP is required for sensitizing cells to lysosphingolipid-induced lysosomal cell death (Stahl-Meyer et al., 2022). Conversely, cAMP signaling has been shown to prevent netosis by inhibiting NOX activity and ROS production through cAMP/PKA pathway (Bokoch et al., 2009), and to prevent cellular senescence via regulation of SIRT1 through cAMP/PKA/CREB pathway (Sung et al., 2020; Zhu et al., 2022). In ferroptosis, PKA activation suppresses mitochondrial activation and upregulates GPX4 via CREB to reduce ROS production and ferroptosis (Ishii et al., 2019), while Epac1 inhibition decreases mitochondrial superoxide levels and subsequently prevents ferroptosis (Musheshe et al., 2022). Therefore, modulation of cAMP signaling pathways bears the potential for targeting various RCDs in AD and PD.

Pharmacologically modulating PKA can result in unintended adverse effects due to its ubiquitous expression in various tissues and cells. To address this issue, alternative strategies that increase the specificity of PKA signaling have been proposed (Insel et al., 2012). Recently, it was found that the type I regulatory subunit of PKA drives the formation of cAMP and PKA condensate, which facilitates cAMP subcellular compartmentation (Zhang et al., 2020). This finding suggests that selectively targeting PKA regulatory or catalytic subunits that are expressed in a specific tissue or subcellular compartment could be a potential strategy to minimize the side effects of PKA activation. The type I-beta and type II-beta regulatory subunits of PKA are promising candidates for modulation due to their selective expression patterns in the brain and lower expression levels in other tissues, and may provide new therapeutic opportunities against RCDs in AD and PD. Additionally, targeting AKAPs such as brain specific AKAP5, or specific phosphorylation targets of PKA can also increase the specificity of PKA signaling. Interestingly, it has been reported that AKAP5 is upregulated in AD patients (Jicha et al., 1999). In addition, AKAP5 has been reported to diminish β-adrenoceptor-induced cAMP signaling (Cong et al., 2001). Targeting of AKAP5 signaling has successfully applied to diminish cardiomyocyte dysfunctioning (Musheshe et al., 2018a; 2018b). By employing these approaches, we hypothesize that it might be possible in the future to achieve targeted activation of PKA signaling while minimizing the potential for off-target effects.

Modulating Epac signaling could be another potential therapeutic strategy against RCDs in neurodegenerative diseases. Indeed, it was reported that Epac1 gene was upregulated whereas Epac2 gene was downregulated in hippocampal and frontal cortex samples of AD patients compared to age-matched controls (Mcphee et al., 2005). Likewise, inhibition of Epac1 largely diminished ROS-related ferroptosis in HT22 hippocampal cells (Musheshe et al., 2022). It is tempting to speculate that an sophisticated interplay between Epac1 and Epac2 may of central importance in the regulation of ROS-driven RCDs in AD and PD. Unlike PKA, Epac2 is specifically expressed in the brain and adrenal glands, and has been implicated in neuronal differentiation and regeneration, thereby potentially reducing side effects in other tissues (Schmidt et al., 2013). Recently, a specific Epac2 activator Sp-8-BnT-cAMPS (“S220”) was developed using structure-guided chemistry, exhibiting 10 times higher potency and 7 times efficacy for Epac2 compared to cAMP (Schwede et al., 2015). Furthermore, a membrane-penetrating version of S220 called S223-AM has been developed recently expected to cross BBB and being therefore more suitable for future treatment opportunities in AD and PD (Xu et al., 2019). Definitely, further research is needed to elucidate the specific mechanisms involved in Epac2-mediated signaling, and its potential interplay with Epac1 against RCDs.

In addition to traditional low molecular weight modulators, PROteolysis TArgeting Chimeras (PROTACs) have shown promising effect in selective degradation of target proteins opening new avenues to specifically inhibit the cAMP pathway. PROTACs are bifunctional molecules with specific ligands for protein of interest and E3 ubiquitin ligase, connected by a linker (Sun et al., 2019). The target protein forms a ternary complex with E3 ubiquitin ligase, and subsequently are ubiquitinated and degraded by ubiquitin proteasome system (Sun et al., 2019). Due to its unique mechanism, PROTACs exhibit higher sensitivity towards drug-resistant targets, which is often induced by exposure to high concentrations of small molecule inhibitors. Furthermore, PROTACs allow direct protein-level knockdown without potential genetic mutations and upregulations compared to other gene-editing techniques (Sun et al., 2019). In the context of AD and PD, PROTACs have reduced pathology and improved cognitive deficits (Konstantinidou et al., 2021; He et al., 2022). In addition, targeting a coactivator of CREB, CREB-binding protein (CBP) that forms a CREB/CBP complex and initiates gene transcription, exhibited a higher antiproliferative effect in myeloma cells compared to traditional inhibitors (Vannam et al., 2021). Next to PROTACs, one might envision to apply the CRISPR/Cas-9 technology to manipulate cAMP pathway. In a recent study, a PDE3A mutant introduced by CRISPR/Cas-9 was shown to provide long-term cardioprotection compared to nonselective PDE3 inhibitors in cardiomyocytes (Ercu et al., 2022). Intriguingly, recent studies point to the usage of molecular glues acting as small molecule mediators that bring two proteins together, regardless of their natural affinity or binding capabilities (Soini et al., 2022). The linker of PROTACs that connects E3 ligase and target proteins is an example of molecular glue. A class of molecular glue are 14-3-3 stabilizers, such as fusicoccin-A and pyrrolidone1, which seem to stabilize the interaction between 14-3-3 and its binding partners (Soini et al., 2022). As outlined in detail above, PKA phosphorylates NOXA1 and recruits 14-3-3, inhibiting the assembly of the NOX1 holoenzyme, ROS production, and ultimately netosis (Bokoch et al., 2009) (Table 1). It is tempting to speculate that 14-3-3 stabilizers strengthen the 14-3-3/NOXA1 complex, and thereby potentially preventing netosis. The inclusion of novel techniques such as PROTACs, CRISPR/Cas-9 and molecular glues will certainly support our future research to further unravel the distinct roles of PKA, AKAPs and Epac’s, their cell-cell type specific interplay to regulate the distinct features of RCDs.
